# Prognosis of Dermatitis Herpetiformis Patients with and without Villous Atrophy at Diagnosis

**DOI:** 10.3390/nu10050641

**Published:** 2018-05-19

**Authors:** Eriika Mansikka, Kaisa Hervonen, Katri Kaukinen, Pekka Collin, Heini Huhtala, Timo Reunala, Teea Salmi

**Affiliations:** 1 Department of Dermatology, Tampere University Hospital, 33521 Tampere, Finland; mansikka.eriika.k@student.uta.fi (E.M.); kaisa.hervonen@staff.uta.fi (K.H.); timo.reunala@uta.fi (T.R.); 2 Celiac Disease Research Center, Faculty of Medicine and Life Sciences, University of Tampere, 33014 Tampere, Finland; katri.kaukinen@staff.uta.fi; 3 Department of Internal Medicine, Tampere University Hospital, 33521 Tampere, Finland; 4 Department of Gastroenterology and Alimentary Tract Surgery, Tampere University Hospital, 33521 Tampere, Finland; pekka.collin@uta.fi; 5 Faculty of Social Sciences, University of Tampere, 33014 Tampere, Finland; heini.huhtala@staff.uta.fi

**Keywords:** dermatitis herpetiformis, coeliac disease, gluten-free diet, small bowel, villous atrophy, prognosis

## Abstract

Dermatitis herpetiformis (DH) is a cutaneous manifestation of coeliac disease. At diagnosis, the majority of patients have villous atrophy in the small bowel mucosa. The objective of this study was to investigate whether the presence or absence of villous atrophy at diagnosis affects the long-term prognosis of DH. Data were gathered from the patient records of 352 DH and 248 coeliac disease patients, and follow-up data via questionnaires from 181 DH and 128 coeliac disease patients on a gluten-free diet (GFD). Of the DH patients, 72% had villous atrophy when DH was diagnosed, and these patients were significantly younger at diagnosis compared to those with normal small bowel mucosa (37 vs. 54 years, *p* < 0.001). Clinical recovery on a GFD did not differ significantly between the DH groups, nor did current adherence to a GFD, the presence of long-term illnesses, coeliac disease-related complications or gastrointestinal symptoms, or quality of life. By contrast, the coeliac disease controls had more often osteopenia/osteoporosis, thyroid diseases, malignancies and current gastrointestinal symptoms compared to the DH patients. In conclusion, villous atrophy at the time of DH diagnosis does not have an impact on the clinical recovery or long-term general health of DH patients.

## 1. Introduction

Dermatitis herpetiformis (DH) is an extraintestinal manifestation of coeliac disease currently affecting approximately 13% of coeliac disease patients [1,2]. DH induces intense pruritus and a symmetrical papulovesicular rash typically on the elbows, knees, and buttocks [3]. Coeliac disease and DH are genetically predisposed by the human leukocyte antigen (HLA) *DQ2* or *DQ8* haplotypes, and exogenous gluten causes an immune response and small bowel mucosal injury in both [4,5]. Furthermore, autoantibodies against endogenous enzyme tissue transglutaminase (TG2) are characteristically present in the serum and the intestine in both conditions [6,7,8,9].

Diagnosis of DH is verified with the detection of pathognomonic granular immunoglobulin A (IgA) deposits in the uninvolved skin by direct immunofluorescence (IF) examination [10]. This IgA is known to target epidermal transglutaminase (TG3) [11], which is considered the autoantigen in DH, while in coeliac disease it is TG2 [6]. In addition to the skin, TG3 antibody response is often present in the sera of DH patients, although TG3 antibodies are occasionally also found in the serum of some coeliac disease patients without DH [12,13,14].

At the time of the DH diagnosis, some degree of small bowel mucosal villous atrophy is known to exist in approximately 75% of patients, but the remainder have normal villous architecture with only coeliac-type inflammation [15,16]. Regardless of the small bowel mucosal alterations, DH patients only rarely present with obvious gastrointestinal symptoms [17,18].

A strict life-long gluten-free diet (GFD) is the mainstay of treatment in both DH and coeliac disease. However, resolution of DH rash can take months or even longer on the dietary treatment, and therefore, DH patients with severe skin symptoms are additionally treated with dapsone medication to control the rash more quickly [3,19]. Coeliac disease and DH both carry an increased risk of concomitant autoimmune conditions such as thyroid diseases and type 1-diabetes; furthermore, the risk of developing non-Hodgkin lymphoma is increased [20,21,22]. Mortality in coeliac disease, but not in DH, has shown to be increased [23]. A GFD is known to have a preventive effect against the development of lymphoma in DH [24], but other than that, previous research about the factors influencing the prognosis of DH is lacking. Currently, it is not known whether DH patients with small bowel villous atrophy at diagnosis have a worse outcome compared to those with normal small bowel mucosa, and furthermore, whether the prognosis of DH patients with villous atrophy is corresponding to that of classical coeliac disease patients. This issue is of importance when necessary investigations, at the time of DH diagnosis, are assessed.

The aim of the current study was to assess whether the presence of villous atrophy at DH diagnosis would affect clinical recovery on a GFD or the long-term prognosis of DH. In addition, DH patients were compared to classical coeliac disease controls with abdominal symptoms at diagnosis and a histologically confirmed diagnosis. The hypothesis of this study was that the presence or absence of villous atrophy at diagnosis would not be an influential factor in the prognosis of DH.

## 2. Materials and Methods

Between 1970 and 2014, a total of 526 DH patients were diagnosed at the Department of Dermatology, Tampere University Hospital. During the study period, all patients with DH living in a defined area around Tampere were diagnosed at this dermatology unit since IF biopsies required for the diagnosis were not performed elsewhere. Each DH patient’s diagnosis was based on the typical clinical picture and the demonstration of granular IgA deposits in skin biopsies [10]. In addition, all diagnosed patients were routinely suggested to undergo gastroscopy and small bowel biopsy obtainment at the time of the diagnosis while on a gluten-containing diet. After diagnosis, a strict GFD was advised to all patients and dapsone was instituted in those with severe skin symptoms. According to routine treatment policies, all patients were followed up at a DH outpatient clinic until the rash had cleared and the dapsone medication could be discontinued. In this study, all DH patients without prior coeliac disease diagnosis (made ≥2 years earlier) diagnosed between 1970 and 2014 and having an available small bowel biopsy result and commencing on a GFD after diagnosis, were included as study patients. Altogether, 352 DH patients fulfilled the inclusion criteria and were included as DH study patients. Further, 248 classical coeliac disease patients with abdominal symptoms at diagnosis and a histologically confirmed diagnosis at Tampere University Hospital during the same time period served as controls.

Data on demographic characteristics, the severity of clinical symptoms and small bowel mucosal histology, and the results of coeliac autoantibodies and hemoglobin values at the time of DH or coeliac disease diagnosis were gathered from the patient records of Tampere University Hospital between March and October 2016. The small bowel biopsy results were graded as subtotal villous atrophy (SVA), partial villous atrophy (PVA), or normal mucosa according to the analysis of the routine pathologist as previously described [16]. In DH patients, the skin symptoms at the time of the diagnosis were graded as mild, moderate, or severe according to the presence of a few, several or many blisters, macular eruptions and erosions. The grading was performed by one dermatologist. In addition, the commencement and duration of dapsone medication after diagnosis was recorded.

Follow-up data were collected using questionnaires (see below for more detail) mailed to all 294 living DH patients fulfilling the inclusion criteria of this study (on December 2015) and the 222 living coeliac disease controls (on May 2016). The final response rate was 62% for the DH patients and 58% for the coeliac disease patients; hence, the follow-up study included 181 DH and 128 coeliac disease patients.

The study protocol and usage of the register-based data were approved by the Regional Ethics Committee of Tampere University Hospital (R15143), and furthermore, informed consent was obtained from each patient participating in the follow-up study.

### 2.1. Questionnaires

The disease-specific questionnaire designed for this study, the Psychological General Well-Being (PGWB) [25] and Gastrointestinal Symptom Rating Scale (GSRS) [26] questionnaires were mailed to the DH and coeliac disease study patients. PGWB and GSRS questionnaires are validated questionnaires, which have been widely applied in previous coeliac disease studies [27,28,29,30,31]. In addition, the DH patients received the Dermatology Life Quality Index (DLQI) questionnaire [32].

The disease-specific questionnaire included both open and multiple-choice questions. The patients were asked about the presence and duration of DH and coeliac disease-related symptoms before and after the diagnosis, the strictness of the GFD, smoking and other lifestyle characteristics, the number of children born, the family history of coeliac disease or DH, and the patient’s current height and weight. Compliance with a GFD was reported as strict diet without dietary lapses, dietary lapses once per month, dietary lapses one to five times per month, or dietary lapses once per week. In addition, the questionnaire included questions about the presence of coeliac disease complications and associated diseases, malignancies, other long-term illnesses, and the regular usage of physician-prescribed medications and over-the-counter (OTC) medications. In the malignancy analysis, non-melanoma skin cancers were excluded, as were excessive trauma fractures in bone fracture analyses.

As previously described, the validated 22-item PGWB questionnaire evaluates self-perceived health-related well-being and distress and includes six dimensions: Anxiety, depressed mood, positive well-being, self-control, vitality, and general health [25]. The total score ranges from 22 to 132, with a higher score indicating better quality of life. The 15-item GSRS questionnaire assesses the severity and existence of gastrointestinal symptoms in five categories: Diarrhea, indigestion, constipation, abdominal pain, and reflux [26]. It uses a seven-point Likert scale for each question: One indicates an absence of symptoms and seven indicates severe symptoms. The DLQI is a 10-item dermatology-specific quality of life instrument. The questionnaire includes six different sections: Symptoms and feelings, daily activities, leisure, work and school, personal relationships, and treatment unit. The scores of all ten questions are calculated together, and the total score varies from a minimum of 0 to a maximum of 30, with a higher score indicating a more impaired life quality [32]. 

### 2.2. Statistical Analysis

A Two-sided chi-squared test was used to compare the categorical variables and a Kruskall–Wallis test was performed to assess differences between the continuous variables. Logistic regression analysis was used to standardize the study groups according to age at the time of the study. Statistical significance was set at *p* < 0.05. The analyses were performed using IBM SPSS Statistics for Windows (Version 23.0., IBM Corp., Armonk, NY, USA).

## 3. Results

### 3.1. DH Patients with Normal Villous Architecture Compared to DH Patients with Villous Atrophy at Diagnosis

Of the 352 DH patients, 98 (28%) had normal villous architecture, and 254 (72%) had small bowel mucosal villous atrophy (PVA or SVA) at the time of the DH diagnosis (Table 1). GFD was not initiated before the diagnosis in study participants. Mean time since the year of DH diagnosis, was 20 years in the DH patients with normal villous architecture and 23 years in the DH patients with villous atrophy, and the difference was not statistically significant. The median age at diagnosis was significantly higher in the DH patients with normal villous architecture compared to the DH patients with villous atrophy (*p* < 0.001, Table 1). At diagnosis, the DH patients with villous atrophy were significantly more often serum coeliac autoantibody-positive compared to the DH patients with normal villous architecture (73% vs. 39%, *p* < 0.001, Table 1). The severity of the DH rash at diagnosis did not differ significantly between the DH groups (*p* = 0.862). Eighty percent of all DH patients used dapsone after the diagnosis. The duration of dapsone usage was longer in the DH patients with normal villous architecture compared to the DH patients with villous atrophy at diagnosis (median 36 vs. 24 months), but the difference was not statistically significant (*p* = 0.097, Table 1).

Of the 181 DH patients with available follow-up data, 39 (22%) had normal villous architecture, and 142 (78%) had villous atrophy at the time of DH diagnosis. The median follow-up time was 20 years in patients with normal villous architecture and 23 years in the DH patients with villous atrophy at diagnosis (Table 2). The presence of gastrointestinal symptoms at diagnosis did not differ between the DH study groups according to the follow-up study questionnaire (*p* = 0.170). At the time of the follow-up study, DH patients with normal villous architecture were significantly older compared to those DH patients who had villous atrophy at diagnosis (Table 2).

The strictness of the GFD and BMI did not differ between the DH study groups at the time of the study (Table 2). Similarly, no significant differences were detected in smoking habits or physical activity: 3% of DH patients without villous atrophy and 13% of patients with villous atrophy at diagnosis were current smokers, and 49% and 69% exercised at least three times a week, respectively.

**Figure 1:**
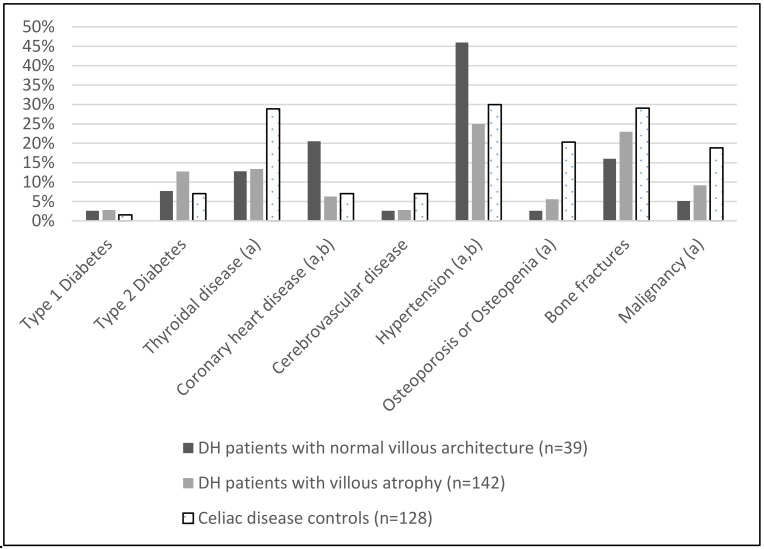
Percentages of dermatitis herpetiformis (DH) patients with normal small bowel mucosal villous architecture and with villous atrophy at diagnosis, and coeliac disease control patients with long-term illnesses or complications at the time of the follow-up study. (a) Statistically significant difference (*p* < 0.05) between the three study groups; (b) statistically significant difference (*p* < 0.05) between DH patients with normal villous architecture and DH patients with villous atrophy at diagnosis.

At the time of the follow-up study, coronary heart disease and hypertension were significantly more common among the DH patients with normal villous architecture compared to the DH patients with villous atrophy at diagnosis (Figure 1); however, after adjustment for the current age, significant differences disappeared (*p* = 0.198, OR = 0.482 and *p* = 0.273, OR = 0.653, respectively). Significant differences were not detected in the presence of type 1- or 2-diabetes, thyroid diseases, cerebrovascular diseases, osteopenia or osteoporosis, or malignancies between the DH study groups (Figure 1). Patients with self-reported bone fractures were slightly more numerous among the DH patients with villous atrophy than among those with normal villous architecture at diagnosis, but the difference was not statistically significant (*p* = 0.321, Figure 1).

Statistically significant differences were not detected in the use of physician-prescribed regular medications between the DH study groups; even the significant difference in the use of statin medication disappeared after adjustment for the current age (OR = 0.479, *p* = 0.88, Table 2). Furthermore, the total amount of used OTC medications was similar in the DH groups; only the usage of vitamin D was more frequent among DH patients with villous atrophy at diagnosis compared to those without villous atrophy (40% vs. 23%, *p* = 0.050).

The presence of gastrointestinal symptoms or the quality of life according to the total or the subscores of the GSRS, PGWB (Table 3) and DLQI questionnaires at the time of the study did not differ between the DH study groups

### 3.2. Comparisons between the DH Patients and the Classical Coeliac Disease Controls

Compared to the DH patients, the coeliac disease controls were more often female (Table 1 and Table 2), and their median diagnostic age was significantly lower compared to DH patients with normal villous architecture at diagnosis (Table 1). 

In the long-term follow-up data, there were no observed differences in current smoking habits or physical activity between the DH patients and the coeliac disease controls. By contrast, the total number of long-term illnesses was found to be higher among the coeliac disease controls compared to the DH patients (Table 2). More specifically, after adjustment for the current age, thyroid diseases (OR = 3.443, *p* = 0.019) and osteopenia or osteoporosis (OR = 14.132, *p* = 0.012) were more common among the coeliac disease controls than among the DH patients (Figure 1). However, the presence of self-reported bone fractures did not differ significantly between the DH study groups and the coeliac disease controls. In the malignancy analysis, the coeliac disease controls outnumbered the DH patients after adjustment for the current age (OR = 6.527, *p* = 0.016) (Figure 1).

In the analysis of regularly used physician-prescribed medications and after adjustment for the current age, the coeliac disease controls were found to use less statin medication compared to the DH patients with normal villous architecture (OR = 0.319, *p* = 0.01). In turn, the total number of regularly used OTCs was higher among the coeliac disease patients compared to the DH patients (*p* = 0.003, Table 2), and specifically the use of calcium (*p* = 0.011) and vitamin D (*p* < 0.001) was more common.

Quality of life measured with the PGWB did not differ between the coeliac disease controls and the DH patients with normal villous architecture at diagnosis, but the coeliac disease controls had significantly lower PGWB general health scores compared to the DH patients with villous atrophy at diagnosis (Table 3). In the GSRS questionnaire, the coeliac disease controls had significantly higher total symptoms gastrointestinal pain and diarrhea scores compared to both DH groups. 

## 4. Discussion

This study demonstrated that the disease severity and the clinical response to a GFD does not differ between DH patients with normal villous architecture and those with villous atrophy at diagnosis. Furthermore, the long-term general health and well-being of DH patients are not influenced by the severity of small bowel mucosal damage at the time of DH diagnosis. The outcomes of the current study were obtained from a large, prospectively collected series of DH patients, all of whom adhered to a GFD treatment. Furthermore, in the present study, the proportions of DH patients with villous atrophy and normal villous architecture were consistent with the findings in earlier DH studies [15,33]. 

In our previous study, the presence of villous atrophy at DH diagnosis was found to be associated with a delayed diagnosis, i.e., the presence of the rash for two years or more before the diagnosis, suggesting that prolonged diagnosis might enable the small bowel mucosal damage to progress [34]. In the current study, the occurrence of villous atrophy did not associate with the severity of the rash or with the presence of gastrointestinal symptoms at diagnosis. The duration of dapsone medication was considered the most reliable method of determining the active period of rash after adherence to a GFD since the majority of patients used dapsone medication, and the medicine was discontinued as early as possible without a relapse in skin symptoms. The median duration of dapsone usage in DH study groups corresponded well with previous GFD treatment studies [35,36], and even though the duration was longer in DH patients with normal small bowel mucosa than in those with villous atrophy at diagnosis, the difference was not statistically significant. Therefore, presence or absence of small bowel villous atrophy at diagnosis seems not to influence the clinical recovery of the DH rash.

The long-term follow-up performed in the present DH patients further demonstrated that small bowel villous atrophy at diagnosis did not have any impact on the presence of long-term illnesses and complications, or long-term quality of life or the presence of persistent gastrointestinal symptoms. Additionally, our previous study showed that the mortality of DH patients with villous atrophy at diagnosis does not differ from that of DH patients with normal villous architecture, and in fact, the mortality of DH patients was shown to be lower than in the general population [37]. Therefore, all these results show that DH patients with and without villous atrophy at diagnosis have a similar good long-term prognosis when they adhere to a GFD.

In contrast to DH, the mortality rate of the patients with coeliac disease are known to be increased compared to the general population [23,38]. Moreover, when the GFD-treated coeliac disease patients in the current study were compared to the DH patient groups, they had significantly more malignancies and long-term illnesses, especially thyroid diseases and osteopenia or osteoporosis. A previous comparison between DH and coeliac disease also showed a higher frequency of diseases of autoimmune origin in patients with coeliac disease [39], but then another study demonstrated that autoimmune diseases were as common among DH patients without classical coeliac disease symptoms than in those DH patients with preceding coeliac disease diagnosis [40]. In the present study, the coeliac disease controls were further found to have worse self-reported general health, and they had more gastrointestinal symptoms at the time of the study compared to the DH patients. These results fit with our recent study that likewise found a better quality of life and fewer gastrointestinal symptoms among long-term treated DH patients compared to treated coeliac disease patients [41].

The results of the present study thus suggest that the prognosis of different phenotypes of coeliac disease diverge and villous atrophy is not the determinative factor in the outcome of DH. Different adherence rates to GFD or varying lifestyle habits did not explain the outcome differences between coeliac disease and DH study patients in this study. One explanation for the different prognosis between coeliac disease and DH might be slightly diverse autoimmune reactions, but this remains to be elucidated in future studies.

In the current study, the median age at diagnosis in DH patients with normal villous architecture was significantly higher compared to DH patients with villous atrophy. We were aware from our earlier long-term DH studies that the age at diagnosis had increased significantly from 1970 onwards [1], and further, that there was a significant trend towards milder villous atrophy [16]. However, in the present study, the time period of DH diagnosis did not differ significantly between DH study groups. Therefore, the divergence in diagnostic periods does not explain the difference in the diagnostic age between DH patients with and without villous atrophy at diagnosis. One explanation might be, however, that older patients are more prone to develop milder small bowel mucosal alterations, e.g., due to divergent immune responses. Previous research shows that older coeliac disease patients are more likely to remain seronegative and further, a trend toward less severe histopathology has been observed with increasing age at the time of coeliac disease diagnosis [42,43]. Nonetheless, these age-related findings detected in coeliac disease and DH should be examined in more detail in further studies.

As a possible limitation of the current study, it must be recognized that the follow-up data were obtained from questionnaires, which might cause selection bias. Recall bias is always a possibility when requiring data from several decades ago. The disease-specific questionnaire used in the study was designed for this particular study and for comparing the results of the study groups, and it has not been used in other disease studies, and it has not been validated. GSRS is not optimized for coeliac disease, but it has been the most commonly used generic questionnaire in coeliac disease studies [28]. PGWB is not a disease-specific instrument, and therefore, it is possible that it might not assess all of the issues that are having an impact on life in DH and coeliac disease patients. GSRS and PGWB questionnaires have not been validated specifically for coeliac disease. Furthermore, all study patients were recruited from the same hospital, and in the future, results from a more comprehensive geographical distribution would be of value. In turn, the major strengths of the study are: A well-defined, prospectively collected DH cohort from a high prevalence area with excellent dietary adherence rates, and the long follow-up time [1]. Moreover, similar large DH studies with knowledge about the diagnostic small bowel mucosal findings and long-term follow-up data consisting of GFD adherence rates have not been performed previously to our knowledge. 

## 5. Conclusions

The major outcome of this study is that skin IgA-IF proven DH patients evincing coeliac-type small bowel mucosal villous atrophy at diagnosis does not differ from DH patients with non-atrophic small bowel mucosa with regard to GFD treatment response, long-term quality of life, or the presence of chronic illnesses or coeliac disease-associated complications.

## Figures and Tables

**Table 1:** Demographic data and disease-related characteristics of 98 dermatitis herpetiformis (DH) patients with normal small bowel villous architecture and 254 DH patients with villous atrophy at diagnosis, and 248 coeliac disease (CD) control patients.

	DH Patients		CD Controls (*n* = 248)	*p*-Value *
	With Normal Villous Architecture (*n* = 98)	With Villous Atrophy (*n* = 254)		
Females; *n* (%)	50 (51)	125 (49)	193 (78)	<0.001
Age at diagnosis; median (range)	52 (3–84)	37 (4–78)	42 (7–75)	<0.001 ^a^
Coeliac autoantibodies ^1^ present in the serum at diagnosis; *n* (%)	28/72 (39)	139/191 (73)	124/148 (84)	<0.001 ^a^
Haemoglobin level at diagnosis ^2^, g/L; median (Q_1_–Q_3_) ^3^	138 (128–148)	136 (129–146)	130 (121–140)	0.057
Dapsone treatment used; *n* (%)	75/93 (81)	191/243 (79)	-	-
Duration of dapsone treatment, months; median (range)	36 (5–324)	24 (2–384)	-	-

* *p*-value measured across the three study groups; ^1^ Transglutaminase 2-, endomysium-, or antireticulin IgA antibodies; ^2^ Statistical analysis was further performed for patients ≥16 years of age and for females and males separately—there were no statistically significant differences between the three groups; ^3^ Interquartile range; ^a^ Statistically significant difference (*p* < 0.001) between DH patients with normal villous architecture and DH patients with villous atrophy.

**Table 2:** Follow-up data of 39 dermatitis herpetiformis (DH) patients with normal villous architecture and 142 DH patients with small bowel mucosal villous atrophy at diagnosis, and 128 coeliac disease (CD) control patients.

	DH Patients		CD Controls (*n* = 128)	*p*-Value *
	With Normal Villous Architecture (*n* = 39)	With Villous Atrophy (*n* = 142)		
Females; *n* (%)	18 (46)	67 (47)	104 (81)	<0.001
Follow-up time, years; median (range)	20 (1–44)	23 (1–42)	18 (6–43)	0.003
Age; median (range)	68 (52–85)	61 (18–96)	65 (34–85)	<0.001 ^a^
BMI, kg/m^2^; median (range)	25 (19–37)	25 (16–38)	26 (15–46)	0.772
Strict adherence to GFD, no dietary lapses; *n* (%)	30 (77)	101 (71)	107 (84)	0.170 ^b^
Number of long-term illnesses; median (range)	1 (0–7)	1 (0–14)	2 (0–9)	<0.001
Number of prescription medications used; median (range)	2 (0–11)	1 (0–18)	3 (0–16)	0.078
Uses statin medication; *n* (%)	14 (36)	21 (15)	15 (12)	0.001 ^c^
Uses antihypertensive medication; *n* (%)	20 (51)	50 (35)	49 (38)	0.188
Uses proton pump inhibitor medication; *n* (%)	5 (13)	16 (11)	16 (13)	0.938
Number of over-the-counter medications used; median (range)	0 (0–5)	1 (0–7)	2 (0–7)	<0.001
Number of children born; median (range)	2 (0–5)	2 (0–6)	2 (0–5)	0.497
First-degree relatives with DH or CD; *n* (%)	13 (33)	53 (37)	55 (43)	0.464

BMI: Body mass index; GFD: Gluten-free diet. * *p*-value measured across the three study groups; ^a^ Statistically significant difference (*p* < 0.001) between DH patients with normal villous architecture and DH patients with villous atrophy at diagnosis; ^b^ *p*-value was tested for categorical variables including categories: strict diet, dietary lapses once per month, dietary lapses 1–5 times/month, dietary lapses once per week; ^c^ Statistically significant difference (*p* = 0.003) between DH patients with normal villous architecture and DH patients with villous atrophy at diagnosis.

**Table 3:** The Psychological General Well-Being (PGWB) and Gastrointestinal Symptom Rating Scale (GSRS) questionnaires’ median and interquartile range (Q1–Q3) results for the gluten-free diet-treated dermatitis herpetiformis (DH) patients with normal villous architecture and with villous atrophy at diagnosis, and the coeliac disease (CD) controls at the time of the follow-up study.

		DH Patients				CD Controls (*n* = 128)		*p*-Value *
		With Normal Villous Architecture (*n* = 39)		With Villous Atrophy (*n* = 142)				
PGWB		median	(Q_1_–Q_3_)	median	(Q_1_–Q_3_)	median	(Q_1_–Q_3_)	
	Total	110	(99–116)	110	(101–117)	106	(96–117)	0.200
	Anxiety	26	(23–27)	26	(23–27)	25	(23–28)	0.891
	Depression	17	(16–18)	17	(16–18)	17	(15–18)	0.587
	Well-being	18	(16–20)	18	(16–20)	18	(16–20)	0.279
	Self-control	16	(15–17)	16	(15–17)	16	(14–17)	0.295
	General health	13	(12–15)	14	(12–16)	13	(11–15)	0.022
	Vitality	20	(17–21)	19	(17–21)	18	(16–20)	0.104
GSRS		median	(Q_1_–Q_3_)	median	(Q_1_–Q_3_)	median	(Q_1_–Q_3_)	
	Total	1.6	(1.3–2.0)	1.7	(1.3–2.3)	2.1	(1.5–4.2)	<0.001
	Diarrhoea	1.0	(1.0–1.7)	1.3	(1.0–2.3)	1.7	(1.0–2.7)	0.006
	Indigestion	1.8	(1.5–2.5)	2.0	(1.5–2.5)	2.0	(1.5–3.0)	0.227
	Constipation	1.7	(1.0–2.3)	1.7	(1.0–2.3)	1.7	(1.0–2.4)	0.482
	Pain	1.3	(1.0–1.7)	1.7	(1.0–2.0)	1.7	(1.3–2.3)	0.007
	Reflux	1.0	(1.0–1.5)	1.0	(1.0–2.0)	1.5	(1.0–2.0)	0.084

* *p*-value measured across the three study groups.
